# Functional assessment of experimental lymphedema: a focused mini-review of animal models and measurement methods

**DOI:** 10.3389/fphys.2026.1912564

**Published:** 2026-10-19

**Authors:** Shao-Yun Hsu, James Brandon Dixon, Zhanna Nepiyushchikh

**Affiliations:** 1 Wallace H. Coulter Department of Biomedical Engineering, Georgia Institute of Technology and Emory University, Atlanta, GA, United States; 2 Division of Reconstructive Microsurgery, Department of Plastic and Reconstructive Surgery, Chang Gung Memorial Hospital, Taoyuan, Taiwan; 3 College of Medicine, Chang Gung University, Taoyuan, Taiwan; 4 George W. Woodruff School of Mechanical Engineering, Georgia Institute of Technology, Atlanta, GA, United States

**Keywords:** animal models, experimental design, functional assessment, lymphatic function, lymphedema

## Abstract

Lymphedema is a chronic progressive disease resulting from impaired lymphatic transport. A wide range of animal models has been developed to study their pathophysiology, yet how well lymphatic function is measured within these models has received comparatively little attention. This mini-review organizes assessment methodologies into three complementary tiers: Tier 1 covers morphological and indirect outcome measures, including volumetric quantification and histological assessment; Tier 2 covers imaging-based approaches, including near-infrared fluorescence lymphangiography, radionuclide lymphoscintigraphy, and magnetic resonance lymphangiography; and Tier 3 covers direct physiological measurement of physical quantities such as intraluminal pressure, lymphatic conductance, and albumin transport rate. Surveying primary studies across seven species, we identify a recurring mismatch between the species most commonly used for lymphedema modeling and those in which the most informative measurements are technically accessible. The most accessible endpoints tend to reflect late-stage tissue consequences rather than early pumping dysfunction, imaging readouts remain constrained to relative dye intensity rather than true physical flow quantities, and direct physiological measurement is sparse and limited to single time points. For acquired secondary lymphedema, the rat hindlimb model paired with quantitative longitudinal imaging and vessel-level analysis offers a balanced platform, whereas genetic mouse models remain preferable for congenital disease and large-animal models for microsurgical and clinical-scale intervention studies; a cross-tabulation by species and measurement tier is provided to guide model and assessment selection.

## 1 Introduction

The lymphatic system is a hierarchical network of vessels and lymphoid organs that complements the cardiovascular system. Its physiological functions can be grouped into four interrelated categories: (1) maintenance of tissue fluid homeostasis by continuous uptake of capillary ultrafiltrate and return of plasma proteins to the venous circulation; (2) immune surveillance and adaptive immunity through delivery of antigens and antigen-presenting cells to draining lymph nodes; (3) absorption and transport of dietary lipids and fat-soluble vitamins via intestinal lacteals; and (4) regulation of interstitial pressure dynamics and macromolecular clearance. Many of these topics have been addressed in recent comprehensive reviews. (Breslin et al., 2018; Oliver et al., 2020; Petrova and Koh, 2020; Davis, 2025) In this article, we focus on how lymphatic function is measured and how those functions relate to lymphedema development. The understanding of lymphatic function has progressed from descriptive morphology to mechanobiological dynamics. Mid-twentieth-century lymphangiography enabled *in vivo* visualization of lymphatic architecture, and the development of glass micropipettes and pressure transducers, together with subsequent *in vivo* cannulation in large-animal models, allowed the first direct measurements of hydrostatic pressure and peripheral lymph flow within individual lymphatic vessels (McHale and Roddie, 1976, 1983; McGeown, 1987). Subsequent refinement of intravital microscopy revealed the lymphangion as the functional contractile unit, in which intrinsic smooth-muscle contractions and coordinated valve gating propel lymph against pressure gradients (Schmid-Schonbein, 1990; Breslin et al., 2018); more recently, servo-null micropipette techniques have extended direct hydrostatic pressure measurement to small-species lymphatic networks (Davis, 2026). The physiological variables that are most biologically meaningful for lymphedema are those describing intrinsic lymphatic pumping: contraction frequency, contraction amplitude, ejection fraction, intraluminal pressure, valve gating thresholds, and lymph flow rate. These parameters together determine whether the lymphatic network can sustain net antegrade transport against interstitial filtration and the hydrostatic load imposed by tissue and gravity (Zawieja, 2009; Scallan et al., 2016; Davis, 2026)When pumping capacity falls below the rate of interstitial fluid generation, fluid accumulates, the vessel wall remodels, vessels dilate, the normal structure of the valves is disrupted and they lose function, and failure of the contractile units propagates, and the disease progresses toward irreversible tissue fibrosis (Weiler et al., 2019; Walker, 2021; Lee and Kim, 2024). Methodologically, however, quantitative measurement of these parameters *in vivo* remains constrained by the small caliber, optical transparency, and exceedingly low flow rates of lymphatic vessels (Munn and Padera, 2014; Sevick-Muraca, 2014). Direct measurements have therefore historically been restricted to large-animal cannulation (McHale and Roddie, 1976, 1983; McGeown, 1987)or controlled ex vivo and *in vitro* preparations such as pressure myography of isolated lymphangions and transmural flow assays of explanted vessels (Chakraborty, 2015; Breslin et al., 2018).

Primary and secondary lymphedema share the final phenotype of impaired lymph transport but arise from distinct initiating mechanisms and therefore require different experimental models. Primary lymphedema results from developmental or inherited abnormalities of lymphatic vessels, valves, or lymphatic patterning and is most directly modeled using genetic or developmental perturbations, including FOXC2 haploinsufficiency and other models that reproduce defined defects in lymphatic development or valve function. Secondary lymphedema, by contrast, follows acquired disruption of an initially formed lymphatic network, most commonly after lymph-node dissection, irradiation, trauma, infection, or other tissue injury (Grada and Phillips, 2017). Stagnant lymph promotes persistent immune-cell accumulation and inflammatory signaling (Brown et al., 2022; Bowman and Rockson, 2024; Lee and Kim, 2024) that can contribute to fibrosis, adipose deposition, lymphatic remodeling, and progressive deterioration of transport (Rockson et al., 2018; Wei et al., 2024). At present, no curative therapy exists; clinical management relies on compression, manual lymphatic drainage, and supportive care, none of which corrects the underlying defect in lymphatic transport (Chang et al., 2016; Lynn, 2023) Lymphovenous anastomosis and vascularized lymph node transfer can physiologically reconstruct the impaired lymphatic system but require advanced microsurgical capability that most patients cannot access. (Grada and Phillips, 2017; Vargo, 2024 Clinical diagnosis still relies on physical findings and indirect quantitative metrics that primarily reflect morphological change rather than intrinsic pumping function (Chang et al., 2016; Grada and Phillips, 2017; Vargo, 2024). Diagnostic criteria commonly incorporate ≥10% volume difference between limbs or circumferential difference ≥2 cm at standardized locations (O'Donnell, 2017; Lymphology, 2020). Functional confirmation is typically obtained via lymphoscintigraphy and near-infrared fluorescence imaging, but no method directly measures contraction frequency, amplitude, pressure, or flow rate in human patients (O'Donnell, 2017; Lymphology, 2020; Vargo, 2024).

To improve diagnosis and treatment, we need a better understanding of the pathophysiology of lymphedema. Therefore, a wide range of animal models have been developed (Podder et al., 2025). Prior reviews have evaluated these models on the basis of induction rate, severity, reproducibility, animal care, and cost (Frueh et al., 2016; Hsu et al., 2022; Podder et al., 2025). However, these evaluations have largely overlooked how well lymphatic function is measured in these models. Increasing evidence indicates that alterations in pumping function precede and predict the onset, progression, and severity of lymphedema (Munn and Padera, 2014; Weiler et al., 2019). By the time edema reaches a clinically detectable threshold, pumping function has often already sustained substantial, or even irreversible, damage (Walker, 2021; Lee and Kim, 2024). Earlier detection is therefore associated with improved outcomes (Donahue, 2015; Thomis, 2023). The purpose of this review is to examine existing animal models of lymphedema, organize current approaches to measuring lymphatic function, and provide practical suggestions for selecting suitable models and functional assessment strategies. In parallel, we identify gaps in current measurement practice and highlight directions for next-generation models and assessment methods.

As a focused mini-review, we do not aim to provide an exhaustive systematic evaluation of all experimental models of lymphatic injury or dysfunction. We do not cover models that create lymphatic injury or functional impairment without detectable swelling. However, such models may provide important information about early or subclinical lymphatic dysfunction. We focus primarily on representative, commonly used animal models in which lymphatic disruption results in an observable lymphedema phenotype, particularly measurable tissue or limb swelling. We organize approaches to measuring lymphatic function into a practical three-tier framework, and provide guidance for matching experimental models with appropriate functional assessment strategies.

## 2 Methodologies for assessing lymphatic function

Across animal models surveyed in this review, methodologies for assessing lymphatic function can be organized into three complementary tiers that differ in their proximity to intrinsic physiology and capacity for direct functional quantification. **Table 1** maps the 70 primary studies surveyed here onto these three tiers, separated by species, showing for each tier which papers fall into the more rigorous sub-criterion (per-animal volumetric cutoff, quantitative temporal readout, or *in vivo* direct measurement) versus the less rigorous alternative.

**Table 1:** Cross-tabulation of 70 lymphedema animal model studies by species and lymphatic function measurement tier sub-criterion.

Tier	Sub-criterion	Mouse	Rat	Rabbit	Large animal
Tier 1	Explicit per-animal volumetric cutoff defined	Jørgensen et al., 2018; Weiler et al., 2019	Nguyen et al., 2023; Xing et al., 2025; Hsu et al., 2026; Sakarya et al., 2022	—	Han et al., 1985
	Volume / circumference measurement, no cutoff	Mendez et al., 2012; Chang et al., 2013; Park et al., 2013; Kimura et al., 2014; Jang et al., 2016; Yoshida et al., 2015; Bramos et al., 2016; Gousopoulos et al., 2016; Gardenier et al., 2017; Gousopoulos et al., 2017, 2017; Iwasaki et al., 2017; Morfoisse et al., 2018; Wiinholt et al., 2019; Wang et al., 2020; Yoon et al., 2020; Hassanein et al., 2021; Hoshino et al., 2023; Yang et al., 2024; Ahmed et al., 2025	Kanter et al., 1990; Cook et al., 1994; Galanzha et al., 2007; Serizawa et al., 2011; Kawai et al., 2014; Yang et al., 2014; Lynch et al., 2015; Matsumoto et al., 2018; Daneshgaran et al., 2019; Triacca et al., 2019; Cheon et al., 2023; Cheon et al., 2024; Cui et al., 2023	Yoon et al., 2003; Kubo et al., 2010; Zhou et al., 2011; Fernández Peñuela et al., 2018; Fernández Peñuela et al., 2019; Campos et al., 2022	Suami et al., 2016; Lähteenvuo et al., 2011; Hadamitzky et al., 2016; Tobbia et al., 2009; Baker et al., 2010; Wu et al., 2014
	Histological assessment (H&E, IHC)	Kriederman et al., 2003; Miteva et al., 2010; Mendez et al., 2012; Zampell et al., 2012; Markhus et al., 2013; Park et al., 2013; Kimura et al., 2014; Jang et al., 2016; Yoshida et al., 2015; Bramos et al., 2016; Geng et al., 2016; Gousopoulos et al., 2016; Gardenier et al., 2017; Gousopoulos et al., 2017, 2017; Iwasaki et al., 2017; Karlsen et al., 2017; Komatsu et al., 2017; Ishikawa et al., 2019; Jørgensen et al., 2018; Morfoisse et al., 2018; Nakajima et al., 2018; Yamaji et al., 2018; Weiler et al., 2019; Wiinholt et al., 2019; Wang et al., 2020; Yoon et al., 2020; Hassanein et al., 2021; Hoshino et al., 2023; Yang et al., 2024; Ahmed et al., 2025	Galanzha et al., 2007; Serizawa et al., 2011; Kawai et al., 2014; Yang et al., 2014; Lynch et al., 2015; Visconti et al., 2016; Matsumoto et al., 2018; Daneshgaran et al., 2019; Cheon et al., 2023a; Cheon et al., 2024; Cui et al., 2023; Nguyen et al., 2023; Xing et al., 2025; Hsu et al., 2026; Sakarya et al., 2022	Yoon et al., 2003; Kubo et al., 2010; Zhou et al., 2011; Fernández Peñuela et al., 2018; Fernández Peñuela et al., 2019	Suami et al., 2013, 2016; Lähteenvuo et al., 2011; Hadamitzky et al., 2016; Tobbia et al., 2009; Baker et al., 2010; Wu et al., 2014
Tier 2	Pattern observation only (stasis, dermal backflow)	Kriederman et al., 2003; Park et al., 2013; Yoshida et al., 2015; Geng et al., 2016; Iwasaki et al., 2017; Komatsu et al., 2017; Ishikawa et al., 2019; Morfoisse et al., 2018; Nakajima et al., 2018; Wang et al., 2020; Hassanein et al., 2021	Lynch et al., 2015; Visconti et al., 2016; Matsumoto et al., 2018; Cheon et al., 2023b; Sakarya et al., 2022	Fernández Peñuela et al., 2018; Fernández Peñuela et al., 2019; Campos et al., 2022	Han et al., 1985; Suami et al., 2013, 2016
	Tracer clearance, intensity quantification, or drainage rate	Proulx et al., 2010; Mendez et al., 2012; Markhus et al., 2013; Kimura et al., 2014; Bramos et al., 2016; Gousopoulos et al., 2017; Jørgensen et al., 2018; Yamaji et al., 2018; Wiinholt et al., 2019; Yoon et al., 2020; Hoshino et al., 2023; Yang et al., 2024; Ahmed et al., 2025	Kanter et al., 1990; Cook et al., 1994; Anthony et al., 1997; Serizawa et al., 2011; Kawai et al., 2014; Yang et al., 2014; Daneshgaran et al., 2019; Triacca et al., 2019; Cui et al., 2023; Nguyen et al., 2023; Xing et al., 2025	Yoon et al., 2003; Jiang et al., 2010	Blum et al., 2010; Lähteenvuo et al., 2011; Hadamitzky et al., 2016; Johnson et al., 2022; Tobbia et al., 2009; Baker et al., 2010; Wu et al., 2014
	Pumping cycle quantification (frequency, amplitude)	Gousopoulos et al., 2016; Gardenier et al., 2017; Gousopoulos et al., 2017; Weiler et al., 2019	Cheon et al., 2024; Hsu et al., 2026	—	—
Tier 3	Direct in vivo measurement of physical quantities (pressure, flow, conductance, albumin transport)	Miteva et al., 2010; Markhus et al., 2013; Karlsen et al., 2017	Galanzha et al., 2007; Lynch et al., 2015	—	Han et al., 1985; Tobbia et al., 2009; Baker et al., 2010
	Ex vivo pressure myography of isolated vessels	Castorena-Gonzalez, 2022	—	—	—

Papers may appear in multiple rows within a tier (e.g., a study performing both volume measurement and histology appears in both Tier 1 rows).

### 2.1 Morphological and indirect outcome measures

Tier 1 encompasses morphological and indirect outcome measures—volumetric quantification by water displacement (Rutkowski et al., 2006; Chang et al., 2013; Gardenier et al., 2017; Iwasaki et al., 2017), caliper or circumferential measurement (Kriederman et al., 2003; Jørgensen et al., 2018); μCT (Yang et al., 2014; Nguyen et al., 2023); magnetic resonance imaging of soft-tissue and fat volumes (Daneshgaran et al., 2019); and immunohistochemical analysis with lymphatic endothelial markers, including LYVE-1 (Kriederman et al., 2003; Miteva et al., 2010), VEGFR-3 (Yoshida et al., 2015; Hoshino et al., 2023), Prox-1 (Park et al., 2013; Bramos et al., 2016), and podoplanin (Mendez et al., 2012; Park et al., 2013). These are the most widely adopted across species owing to their technical accessibility and reproducibility. They reliably capture the downstream consequences of lymphatic insufficiency but are inherently limited in detecting early or subclinical dysfunction, and changes may lag substantially behind the onset of functional impairment (Weiler et al., 2019; Lee and Kim, 2024).

### 2.2 Imaging-based functional assessments

Tier 2 comprises imaging-based functional assessments, including near-infrared fluorescence (NIR) lymphangiography with indocyanine green (Suami et al., 2016; Hassanein et al., 2021; Nguyen et al., 2023), radionuclide lymphoscintigraphy (Kanter et al., 1990; Yang et al., 2014; Jørgensen et al., 2018), magnetic resonance lymphangiography with gadolinium-based contrast agents (e.g., Gd-BOPTA) (Jiang et al., 2010), whole-body fluorescence platforms such as the *In Vivo* Imaging System (IVIS), and Evans blue dye lymphangiography (Proulx et al., 2010; Yamaji et al., 2018). These modalities bridge morphological endpoints and direct physiological measurement by enabling real-time or time-resolved visualization of lymphatic transport *in vivo* (Munn and Padera, 2014; Sevick-Muraca, 2014). NIR imaging in permits relative-quantitative analysis of contractile dynamics via packet-based metrics, including contraction frequency, amplitude, and transport (pumping) score, derived from time-resolved changes in fluorescence intensity rather than static images (Gousopoulos et al., 2016; Weiler et al., 2019; Cheon et al., 2023a). However, because the fraction of dye taken up by lymphatics from an interstitial injection cannot be measured directly, fluorescence intensity readouts remain relative rather than absolute, and conventional Tier 2 modalities do not yield absolute flow values.

### 2.3 Direct physiological measurement

Tier 3 consists of direct physiological assessments, including ex vivo pressure myography with cannulated lymphangions, *in situ* lymphatic conductance measurement, interstitial fluid pressure quantification (Markhus et al., 2013), transmural flow assays (Chakraborty, 2015; Breslin et al., 2018), and *in vivo* lymph cannulation (Han et al., 1985; Tobbia et al., 2009; Baker et al., 2010). Ex vivo pressure myography of isolated cannulated segments enables precise characterization of valve competence, back-leak pressure, gating thresholds, contraction frequency, amplitude, and ejection fraction at the lymphangion level (Castorena-Gonzalez, 2022). *In situ* lymphatic conductance quantifies the volume of interstitial fluid drained per unit tissue, time, and applied pressure, separating lymphatic uptake from surrounding tissue hydraulic conductivity (Miteva et al., 2010). Transmural flow assays in explanted vessels examine how pressure gradients across the vessel wall regulate endothelial permeability and immune-cell migration (Chakraborty, 2015; Breslin et al., 2018). In larger animals, *in vivo* cannulation of thoracic ducts or peripheral collecting vessels enables direct measurement of lymph flow rate, intraluminal pressure dynamics, and lymph composition (McHale and Roddie, 1983; Han et al., 1985; McGeown, 1987; Tobbia et al., 2009). The principal limitation of this tier is technical: the methods require specialized instrumentation, surgical expertise, and, frequently, transection of the vessel, thereby restricting use to mechanistic studies and limiting longitudinal monitoring within the same subject. Current approaches, therefore, provide only limited longitudinal characterization of lymphatic function across healthy, disease-progression, and treated states.

## 3 Animal models of lymphedema: a contextual summary

Several systematic and narrative reviews have examined the comparative characteristics of lymphedema animal models, including cost, reliability, technical accessibility, induction rate, clinical relevance, and histopathological fidelity to human lymphedema (Frueh et al., 2016; Hsu et al., 2022; Podder et al., 2025). These dimensions have been thoroughly covered; we provide only a concise summary here to frame the subsequent analysis. Non-mammalian species, including zebrafish, differ markedly from humans in anatomy and physiology and are rarely adopted as contemporary lymphedema models (Podder et al., 2025). Among mammals, the mouse remains the most widely used species owing to its low cost, mature genetic tools, and broad availability of primary and secondary models (Frueh et al., 2016; Hsu et al., 2022; Podder et al., 2025). Well-established transgenic lines, including FOXC2 haploinsufficient (Kriederman et al., 2003) and K14-VEGFR3-Ig mice (Markhus et al., 2013), recapitulate key aspects of human primary lymphedema. The principal secondary mouse model is the tail model, which, however, diverges substantially from human limb lymphedema in anatomy, pathophysiology, and histology, and is further limited by spontaneous resolution (Rutkowski et al., 2006; Frueh et al., 2016; Hsu et al., 2022; Podder et al., 2025). Mouse hindlimb lymphedema, established via popliteal lymphadenectomy or genetically inducible lymphatic ablation (Huang et al., 2016; Iwasaki et al., 2017; Hoshino et al., 2023), offers closer anatomical correspondence to human limb lymphedema than the tail model but remains constrained by small vessel caliber. Rat models—typically induced by hindlimb or forelimb lymphadenectomy with adjuvant irradiation—offer closer anatomical and pathological correspondence to human secondary lymphedema and more reliably yield persistent swelling (Frueh et al., 2016; Hsu et al., 2022; Podder et al., 2025). Large animal models, including rabbit, dog, pig, sheep, and nonhuman primate models, most closely resemble human lymphatic anatomy and the effects of gravity, but are constrained by cost and lower throughput (Hsu et al., 2022). Notably, porcine models have repeatedly failed to produce meaningful limb volume change despite extensive surgical disruption (Blum et al., 2010; Lähteenvuo et al., 2011; Hadamitzky et al., 2016), whereas rabbit, dog, sheep, and nonhuman primate models have documented measurable edema (Han et al., 1985; Tobbia et al., 2009; Baker et al., 2010; Yang et al., 2014; Fernández Peñuela et al., 2019).

## 4 Observations on current measurement practice

Having outlined the three-tier framework and summarized the prevailing consensus on animal models, we now examine the intersection of these two dimensions (**Table 1**). The observations below emerged from a focused survey of the 70 lymphedema-relevant primary studies covered by this review.

### 4.1 Tier 1 assessment: divergent practice between primary and secondary models

The mouse is the only mammalian species with well-established primary lymphedema models (Podder et al., 2025). Because the phenotype arises from the genetic manipulation, the induction rate is effectively 100% in animals confirmed to carry the transgene (Podder et al., 2025). As a result, studies using primary mouse lymphedema models confirm lymphedema by genotype or morphological inspection rather than by Tier 1 measurements, particularly the degree of swelling, to determine whether individual animals have developed lymphedema (Kriederman et al., 2003; Markhus et al., 2013). This approach is reasonable, but it also means that data on the main symptom of primary lymphedema, swelling, and on disease severity are largely absent from these studies.

From the rat onward, transgenic models are not routinely available. Most secondary lymphedema is induced by surgical disruption of lymphatic drainage, frequently combined with adjuvant irradiation, with overall induction rates of approximately 60–80% (Yang et al., 2014; Jørgensen et al., 2018; Nguyen et al., 2023; Ahmed et al., 2025). Quantitative confirmation of persistent swelling via Tier 1 measurement is therefore essential to verify successful model establishment. This concern has been articulated in prior reviews. Frueh et al. (2016) questioned whether results from animals showing a 5% versus a 20% increase in limb circumference or a 5% increase in volume can be meaningfully compared, and Hsu et al. (2022) identified the absence of agreed-upon measurement standards as a major limitation (Frueh et al., 2016; Hsu et al., 2022). What has not previously been quantified is the scale of the gap between recommendation and practice. Among the 62 surgical lymphedema model studies surveyed here, only 7 (11.3%) define an explicit per-animal volumetric cutoff for model establishment: 2 of 27 mouse studies (7.4%; Jørgensen et al. ≥ 20% volumetric difference; Weiler et al. ≥20% circumference stratification) (Jørgensen et al., 2018; Weiler et al., 2019), 4 of 18 rat studies (22.2%; Nguyen et al. at ≥5% by micro-CT volume; Xing et al. at ≥1 mL bilateral difference; Hsu et al. at ≥5%; Sakarya et al. at ≥5% by micro-CT volume) (Sakarya et al., 2022; Nguyen et al., 2023; Xing et al., 2025; Hsu et al., 2026), and 1 of 10 large-animal studies (10%; Han et al., at ≥1 cm circumferential difference) (Han et al., 1985). Three additional studies define non-volumetric cutoffs—durational, qualitative, or functional (Zhou et al., 2011; Suami et al., 2013; Hadamitzky et al., 2016) —bringing the total number of studies that apply any explicit per-animal criterion to 10. Notably, volumetric measurement is performed in most studies—approximately 96% of mouse, 88% of rat, and 88% of large-animal studies report some form of limb, tail, paw, or circumference measurement. MRI-based volumetric assessment was used in only 1 of 70 studies, (Daneshgaran et al., 2019) reflecting cost and access constraints rather than lower accuracy. Rat and large-animal studies apply an explicit cutoff more often than mice (22.2% and 10% vs. 7.4%; **Table 1**), likely because surgical induction, unlike genetic induction, requires a threshold to distinguish true lymphedema from transient postoperative edema. The deficit is therefore not an absence of measurement but the absence of a pre-specified threshold (**Table 1**).

Histological assessment constitutes the second major component of Tier 1 and is performed in the majority of studies across all species: approximately 91% of mouse, 79% of rat, 71% of rabbit, and 70% of large-animal studies report histological endpoints. (**Table 1**) Unlike volumetric measurement, histology does not require a pre-specified threshold to yield interpretable data, as it characterizes tissue-level results of lymphatic failure, including dermal thickening, adipose deposition, fibrosis, and inflammatory infiltration, which are qualitatively distinct from normal tissue. (Gardenier et al., 2017; Yamaji et al., 2018) Immunohistochemical analysis with lymphatic endothelial markers, particularly LYVE-1 (used in 24 of the 70 studies surveyed here) (Kriederman et al., 2003; Park et al., 2013), podoplanin (Mendez et al., 2012; Bramos et al., 2016), and VEGFR-3 (Yoshida et al., 2015; Hoshino et al., 2023), further allows quantification of lymphangiogenesis and lymphatic remodeling. In this respect, histology complements volumetric measurement, which captures the fluid and fat accumulations consequences of lymphatic failure; histology captures the structural and cellular sequelae. Across the studies reviewed, however, the choice of histological markers and the criteria used to score or quantify tissue changes vary substantially between laboratories, and no consensus panel or standardized scoring system has been adopted, limiting comparability in a manner analogous to the volumetric cutoff problem.

### 4.2 Tier 2 imaging predominantly captures lymphatic consequences, not contractile function

Across the 61 studies that employed Tier 2 imaging, the large majority relied on pattern observation alone (22 studies) or on bulk clearance and intensity quantification (33 studies), both of which capture the downstream consequences of lymphatic failure rather than changes in lymphatic pumping function during lymphedema development. Only 6 studies (10%) quantified collecting vessel contraction frequency, amplitude, and pumping score from real-time imaging — and all 6 were conducted in rodents: 4 in mouse tail models (Gardenier et al., 2017; Gousopoulos et al., 2017a, 2017b; Weiler et al., 2019) and 2 in rat limb models (Cheon et al., 2023b; Hsu et al., 2026). No rabbit or large-animal study achieved this level of functional resolution.

In reviewing the imaging data across these studies, a consistent qualitative observation emerges: NIR and fluorescence images from rat limb models are generally clearer than those from mouse models, and the contrast between lymphedema and control limbs is more apparent - a difference that becomes particularly pronounced in pumping function quantification, where the two rat studies demonstrate a more distinct separation in contraction amplitude and transport metrics than the four mouse tail studies, in which the dynamic range of the signal is comparatively compressed.

### 4.3 Tier 3 direct measurement: sparse, species-constrained, and limited to single time points

Among the 70 studies surveyed, direct *in vivo* measurement of physical lymphatic quantities was performed in only 7 studies, with 4 in rodents and 3 in large animals, and ex vivo pressure myography was performed in 1 additional study. This represents the fewest studies across all three tiers.

In rodent models, Miteva et al. measured lymphatic conductance in the mouse tail *in vivo* (Miteva et al., 2010); Markhus et al. quantified interstitial fluid pressure and lymph flow in a transgenic mouse model of primary lymphedema (Markhus et al., 2013); Galanzha et al. measured lymph flow velocity, contraction frequency, and valve activity in rat mesenteric lymphatics using intravital microscopy (Galanzha et al., 2007); and Lynch et al. quantified dextran fluid drainage volume from the rat foreleg (Lynch et al., 2015). In large-animal models, Han et al. directly measured intraluminal lymphatic and venous pressures in canine lymphedema limbs, reporting that lymphatic pressure in lymphedema limbs (10.63 ± 8.61 mm Hg) was significantly higher than in normal limbs (2.77 ± 1.02 mm Hg; p < 0.01) (Han et al., 1985); Tobbia et al. quantified the mass transport rate of radiolabeled human serum albumin from peripheral lymphatics to the systemic circulation in sheep following popliteal lymph node excision (Tobbia et al., 2009); and Baker et al. employed a similar prenodal cannulation approach (Baker et al., 2010). Historical cannulation studies in sheep further demonstrate the feasibility of measuring intraluminal pressure and outflow (McHale and Roddie, 1983).

A shared limitation across all Tier 3 studies is the restriction to a single measurement time point. No Tier 3 study in this survey conducted repeated direct measurements of physical quantities in the same animal over the course of disease progression. This stands in marked contrast to Tier 1 and Tier 2 studies, in which longitudinal measurements in the same animal over the full course of disease development are frequently used.

### 4.4 Selecting a model by biological question

There is no universally ideal species or induction approach for every lymphedema study. Choosing a model should begin with a clear understanding of the underlying disease mechanism and the specific functional aspects to be evaluated. In cases of congenital or primary lymphedema, mouse models with defined genetic backgrounds offer the closest match to human mechanisms, since the primary cause is developmental. These models are particularly valuable for their genetic precision, accessibility to lineage tracing and immune studies, and suitability for high-throughput experiments. However, they are limited by the small size of lymphatic vessels and constraints on direct pressure and flow monitoring.

For studies focusing on secondary lymphedema resulting from cancer treatment, limb models are more suitable, as they mimic the acquired blockage of lymphatic flow. Mouse hindlimb models are especially helpful when the research emphasizes immune responses, genetic alterations, or rapid therapeutic screening. When experiments require ongoing limb swelling, repeated near-infrared imaging of lymphatic transport, or microsurgical procedures, rat models of the hindlimb or forelimb offer a balanced option. Larger animal limb models provide the most realistic scale for reconstructive techniques, direct pressure measurement, and device evaluation, but their high expense, space requirements, ethical considerations, and lower throughput mean they are best reserved for targeted translational research rather than broad exploratory studies.

Measurement strategies should be chosen to fit the specific research hypothesis. Basic measurements (Tier 1) are appropriate when the main focus is on establishing the model or assessing swelling, fibrosis, fat accumulation, or structural changes. More advanced monitoring (Tier 2) is recommended when it is important to track lymphatic transport or vessel pumping over time in the same subject. The most technical measurements (Tier 3) become necessary if the research question involves precise physical parameters such as pressure inside vessels, conductance, valve function, or overall lymph flow. When possible, using multiple measurement approaches together is best, since changes in tissue structure, tracer movement, and lymphatic function may not always align throughout the course of disease and treatment. **Figure 1** summarizes this decision-making framework, providing a visual guide from research question to model and measurement selection.

**Figure 1:**
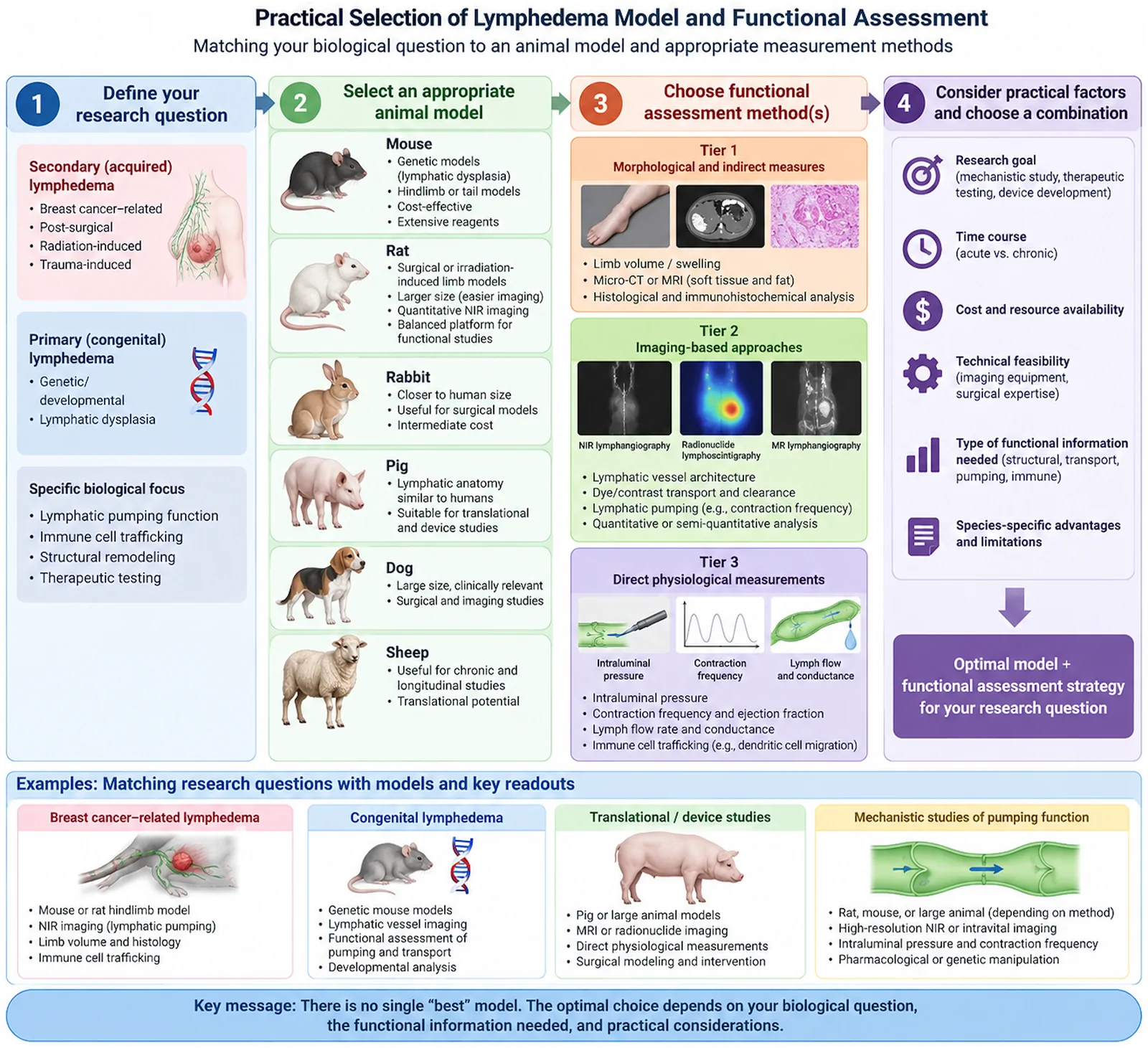
Practical selection of lymphedema model and functional assessment. Matching your biological question to an animal and appropriate measurement methods.

## 5 Discussion

A fundamental constraint shapes how lymphatic function can be assessed in animal models: the most accessible endpoints tend to be the least informative about the disease process, while the most informative are the least accessible. Limb swelling and histological change are themselves consequences of lymphatic dysfunction, but both are late manifestations, emerging only after lymphatic pumping capacity has been overwhelmed for some time and secondary tissue responses have accumulated (Weiler et al., 2019; Lee and Kim, 2024). Because they are temporally distant from the initial injury, these endpoints offer limited insight into the onset and early progression of lymphedema.

Imaging mitigates this delay but depends on injected contrast agents for a physical reason: lymph is optically transparent, contains few scattering cellular elements, and flows at velocities often below the detection threshold of label-free methods such as Doppler-based instrumentation (Munn and Padera, 2014; Sevick-Muraca, 2014). This is why imaging readouts reflect dye intensity rather than a true physical quantity. Emerging modalities, including Doppler optical coherence tomography, which has demonstrated label-free quantification of lymph flow velocity and volumetric flow rates in collecting vessels of living animals (Blatter et al., 2016), and photoacoustic imaging with lymphatic-absorbing contrast agents (Martel et al., 2014), may eventually enable more direct assessment of lymphatic flow, though their application to lymphedema models remains limited. Tier 3 methods circumvent the limitation by measuring physical quantities more directly, through two strategies with different trade-offs. Optical intravital approaches (Galanzha et al., 2007) are comparatively non-disruptive but confined to anatomical sites of limited clinical relevance, whereas cannulation can record intraluminal pressure and flow rate directly in large animals (McHale and Roddie, 1983; Han et al., 1985; McGeown, 1987). Given the effort required to establish *in vivo* cannulation, however, restricting the readout to labeled albumin transport leaves much of the platform’s potential unrealized; the same preparation could simultaneously record intraluminal pressure dynamics, vessel diameter, and lymph composition. Such multi-parameter recordings would provide the boundary-condition data required for computational fluid-dynamic modeling of lymphatic transport in disease.

These constraints interact with species choice to produce a recurring mismatch between the species in which lymphedema is most commonly modeled and those in which the most meaningful measurements are feasible. The mouse is the most accessible platform but constrains the quantitative information extractable from imaging; the rat permits quantitative imaging-based analysis but cannot accommodate *in vivo* cannulation; large animals permit direct cannulation yet are used in too few studies to realize their potential. A further irony compounds this at each tier. Tier 1 measurement is most essential where induction is least reliable, yet the mouse, where induction is most reliable but secondary pathology least representative, is where Tier 1 reporting is most often omitted. Tier 2 quantification is most achievable in the rat, yet qualitative mouse tail imaging is still widely accepted as functional evidence. Tier 3 is most informative in large animals, yet too few such studies exist for the tier to be more than marginally exploited.

Taking these considerations together, no single model is optimal across all research contexts. For acquired secondary lymphedema, the rat surgical or irradiation-induced limb model emerges as arguably one of the more balanced platforms when quantitative longitudinal imaging and vessel-level analysis are required, whereas genetic mouse models remain preferable for studying congenital lymphedema, and large-animal models are better suited to microsurgical intervention and clinical-scale procedures. Focusing on the rat limb model specifically, and based on the characteristics compiled in prior systematic reviews, including cost, accessibility, reproducibility, induction reliability, and clinical fidelity (Frueh et al., 2016; Hsu et al., 2022; Podder et al., 2025), it is large enough to permit quantitative imaging yet far more accessible than large-animal models; the porcine platform, by comparison, has yielded only an acute cannulation assay rather than a chronic disease model (Johnson et al., 2022). Examined through the lens of functional measurement, the priorities for future work become clear. At Tier 1, the field would benefit from consensus standards for quantifying swelling and scoring immunohistochemical change. At Tier 2, where rodent imaging protocols are already well established, two studies have shown that real-time pumping cycle parameters can be extracted from intensity changes over time (Cheon et al., 2023a; Hsu et al., 2026), indicating that pumping function can be approximated even within the dye-intensity constraints of imaging. **Figure 1** and **Table 1** together are intended to support this kind of decision-making, allowing investigators to pair an appropriate animal model with a functional measurement and to locate corresponding protocols for reference.

## 6 Conclusion

No current animal model allows direct, longitudinal measurement of lymphatic pumping function in a clinically relevant setting. Weighing the available models against the measurement methods they support, the rat hindlimb model paired with quantitative longitudinal imaging appears best suited to acquired secondary lymphedema, genetic mouse models remain the preferred choice for congenital lymphedema, and large-animal models are best reserved for microsurgical and clinical-scale intervention studies. Until techniques for longitudinal, non-disruptive measurement of lymphatic flow in diseased animals mature, the pathophysiology of lymphedema as a disease of lymphatic transport is likely to remain incompletely characterized.
